# Double‐Bundle Artificial Medial Patellofemoral Ligament Reconstruction Using a Femoral Suture‐Sliding Anchor and Patellar Staged Tension Adjustment

**DOI:** 10.1002/atn2.70004

**Published:** 2026-04-28

**Authors:** Ryo Sasaki, Takahiro Okawa, Taichi Nishimura, Teppei Hayashi, Kazuya Kaneda, Masaki Nagashima, Hideo Morioka

**Affiliations:** ^1^ Department of Orthopaedic Surgery NHO Meguro‐ku Tokyo Japan; ^2^ Department of Orthopaedic Surgery Keio University School of Medicine Shinjuku Tokyo Japan; ^3^ Department of Orthopaedic Surgery International University of Health and Welfare Mita Hospital Minato‐ku Tokyo Japan

## Abstract

Double‐bundle medial patellofemoral ligament reconstruction with artificial ligaments is a minimally invasive surgical option for recurrent patellar dislocation. However, it carries a risk of over‐tensioning, which may lead to restricted patellar mobility and anterior knee pain. Here, we describe a technique that combines an artificial ligament with a femoral suture‐sliding anchor and double‐tunnel patellar fixation. This construct enables staged tension adjustment, thereby mitigating the risk of over‐tensioning. By introducing the adjustability of the artificial double‐bundle graft at the patellar site, this method offers a reproducible means of fine‐tuning graft tension and avoiding over‐constraint in double‐bundle artificial medial patellofemoral ligament reconstruction.

## Graphical abstract

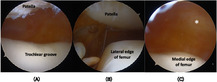

**VIDEO 1:** Medial patellofemoral ligament (MPFL) reconstruction is an effective surgical option for treating recurrent patellar dislocation. In particular, reconstruction using artificial ligaments allows for a minimally invasive procedure with strong initial fixation. However, artificial ligaments are stiffer than autografts, raising concerns about the risk of over‐tensioning. This technique incorporates staged tension adjustment and a femoral suture‐sliding anchor to mitigate this risk. The patient is a 25‐year‐old man who experienced a second episode of right patellar dislocation 3 months prior. Radiography reveals right patellar instability. Under general anesthesia, the patient is placed in the supine position, with the leg secured in a standard leg holder to allow full ROM. Physical examinations are performed under anesthesia to evaluate patellar instability, tracking, and lateral retinacular tightness. Arthroscopy reveals patellofemoral incongruence, patellar maltracking, and laxity of the MPFL region. For femoral fixation, a 1‐ to 2‐cm incision is made at Schöttle's point under fluoroscopic guidance (BEAR Medic). A 2.4‐mm guide pin is inserted, followed by sequential drilling and dilation (all instruments: Smith & Nephew Endoscopy). A 5.5‐mm HEALICOIL anchor with ULTRATAPE (Smith & Nephew Endoscopy) is then placed, and smooth sliding of the ULTRATAPE is confirmed. For patellar fixation, a 2‐ to 3‐cm incision is made along the medial patellar border. The medial retinaculum is incised, and the vastus medialis obliquus is identified. The ULTRATAPE is passed extra‐articularly through the MPFL layer to the patellar incision site. Two patellar bone tunnels are then created under fluoroscopic guidance: a proximal tunnel at the proximal one‐quarter position of the patella and a distal tunnel at the midpoint of the patella Both tunnels are positioned centrally in the anteroposterior plane. The free ends of the ULTRATAPE are advanced laterally using a passing pin (Smith & Nephew Endoscopy). Staged tension adjustment is then performed. The proximal tunnel is first secured with a 5 × 20‐mm BIOSURE screw (Smith & Nephew Endoscopy), and smooth sliding of the double‐bundle construct is confirmed. The strand exiting the distal tunnel is temporarily secured using a Kocher clamp while the knee was flexed and extended. Patellar stability in extension and the absence of over‐constraints beyond 60& flexion are verified. Once optimal tension is achieved, the ULTRATAPE is fixed at the distal tunnel with another BIOSURE screw. Finally, arthroscopy is performed to confirm improved patellar tracking and appropriate MPFL tension. This technique provides a reproducible and safe approach for double‐bundle artificial MPFL reconstruction, offering intraoperative flexibility in tension adjustment and reducing the risk of over‐constraints. Video content can be viewed at https://doi.org/10.1002/atn2.70004.

The medial patellofemoral ligament (MPFL) is the primary restraint against lateral patellar displacement.^1^ Double‐bundle MPFL reconstruction has been advocated to reproduce the fan‐shaped anatomy of the MPFL. Artificial ligament‐based MPFL reconstruction is a minimally invasive technique that provides a strong initial fixation and is particularly useful in skeletally mature patients.^2^, ^3^, ^4^, ^5^ However, compared with autografts, artificial ligaments lack elasticity, and their greater stiffness increases the risk of over‐tensioning, which may result in anterior knee pain, delayed rehabilitation, or degenerative changes.^4^, ^6^ This technical note describes a reconstruction method that uses a femoral suture‐sliding anchor and staged patellar fixation with double tunnels. This technique allows intraoperative adjustment and correction, thereby addressing the critical issue of over‐tensioning in artificial MPFL grafts. Furthermore, in cases of postoperative over‐tensioning, the suture‐sliding property of the femoral anchor may permit graft mobility and help alleviate excessive tension (Video 1).

## SURGICAL TECHNIQUE

### Surgical Indication

This technique is indicated for patients with lateral patellar instability in the absence of pronounced osseous abnormalities. The contraindications include severe trochlear dysplasia (Dejour type D), advanced patellofemoral osteochondral pathology, and skeletal immaturity. In patients with structural abnormalities, such as increased tibial tubercle‐trochlear groove distance (>20 mm), additional osseous realignment procedures should be considered in conjunction with MPFL reconstruction.

### Physical Examination and Arthroscopic Evaluation Under Anesthesia

**FIGURE 1:**
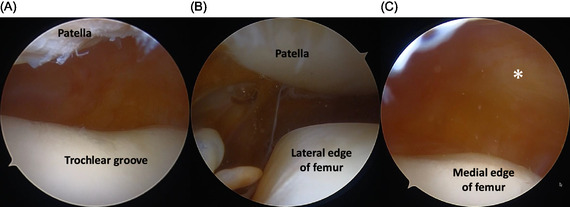
Preoperative arthroscopic evaluation (right knee, anterolateral portal views). The patellofemoral congruence is poor (A), with lateral displacement of the patella (B) and a relaxed medial patellofemoral ligament region (white asterisk) (C).

Under general anesthesia, the patient is placed in the supine position with the leg secured in a standard leg holder, allowing for full range of motion (ROM). Physical examination under anesthesia is routinely performed to evaluate patellar tracking and lateral retinacular tightness. Subsequently, diagnostic arthroscopy is performed to evaluate intra‐articular pathology, patellofemoral congruence, and laxity of the MPFL region (Figure 1). If lateral retinacular tightness is present, lateral retinacular release is performed.

### Femoral Incision and Femoral Suture‐Sliding Anchor Placement

**FIGURE 2:**
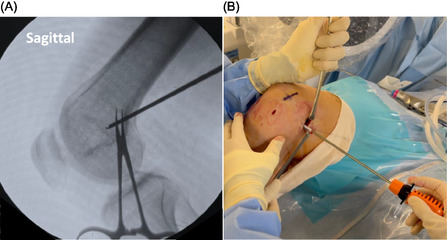
Femoral suture‐sliding anchor placement (right knee, sagittal fluoroscopic and medial views). A 1‐ to 2‐cm incision is made over Schöttle's point, identified under fluoroscopic guidance using a medial patellofemoral ligament guide (BEAR Medic). A 2.4‐mm guide pin is inserted to Schöttle's point under sagittal fluoroscopic guidance (A), followed by EndoButton Drill and sequential dilation. A 5.5‐mm HEALICOIL anchor with ULTRATAPE is inserted (B) (all instruments: Smith & Nephew Endoscopy).

**FIGURE 3:**
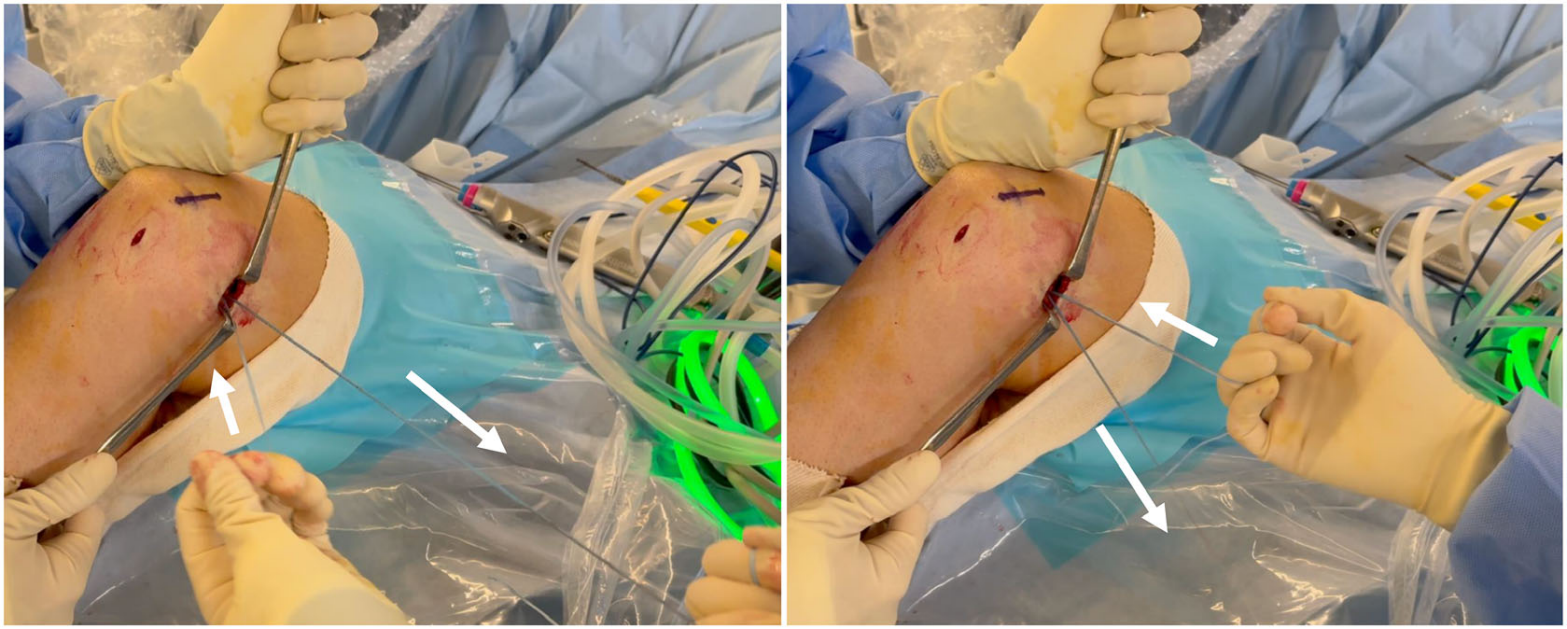
Confirmation of smooth sliding of the ULTRATAPE (right knee, medial views). After placement of the HEALICOIL anchor, both ends of the ULTRATAPE are alternately pulled by hand (arrows) to confirm smooth sliding through the anchor.

A 1‐ to 2‐cm incision is made over Schöttle's point, identified under fluoroscopic guidance using an MPFL guide (BEAR Medic Corp., Tokyo, Japan). After a 2.4‐mm guide pin (Smith & Nephew Endoscopy, Andover, MA) is inserted (Figure 2A), drilling is performed with the 4.5‐mm EndoButton Drill (Smith & Nephew Endoscopy), followed by sequential dilation using the HEALICOIL REGENESORB Dilator (Smith & Nephew Endoscopy). A 5.5‐mm HEALICOIL REGENESORB Suture Anchor with ULTRATAPE Suture (Smith & Nephew Endoscopy) is inserted (Figure 2B), and smooth sliding of the ULTRATAPE is verified (Figure 3).

### Patellar Incision and ULTRATAPE Passage Within the MPFL Layer

**FIGURE 4:**
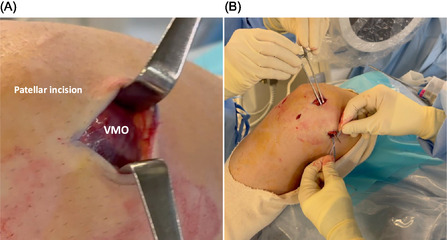
Patellar incision and ULTRATAPE passage within the medial patellofemoral ligament layer (right knee, medial views). A 2‐ to 3‐cm medial patellar incision is made, the medial retinaculum (first layer) is incised, and the vastus medialis obliquus (VMO) is identified (A). The two free ends of the ULTRATAPE are then passed extra‐articularly (second layer) from the femoral incision to the patellar incision (B).

A 2‐ to 3‐cm longitudinal incision is made along the medial patellar border. The medial retinaculum (first layer) is incised, and the vastus medialis obliquus is identified (Figure 4A). The 2 free ends of the ULTRATAPE are then passed extra‐articularly (second layer) from the femoral incision to the patellar incision (Figure 4B).

### Patellar Bone Tunnel Creation and ULTRATAPE Passage

**FIGURE 5:**
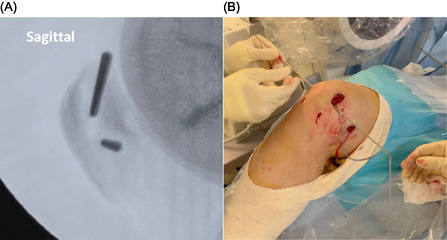
Patellar bone tunnel creation and ULTRATAPE passage (right knee, sagittal fluoroscopic and medial views). Under sagittal fluoroscopic lateral view, guide pins are placed at the proximal one‐quarter (proximal tunnel) and midpoint (distal tunnel) positions of the patella. Both guide pins are positioned centrally in the anteroposterior plane (A). Bone tunnels are created using an EndoButton Drill, and the free ends of the ULTRATAPE are passed to the lateral side of the patella using a passing pin (B).

The periosteum on the medial patellar surface is incised to expose the medial edge. Under fluoroscopic lateral view, 2.4‐mm guide pins are placed at the proximal one‐quarter (proximal tunnel) and midpoint (distal tunnel) positions of the patella. Both guide pins are positioned centrally in the anteroposterior plane (Figure 5A). Bone tunnels are created using an EndoButton Drill. The free ends of the ULTRATAPE are passed to the lateral side of the patella using a passing pin (Figure 5B).

### Staged Tension Adjustment of the MPFL

**FIGURE 6:**
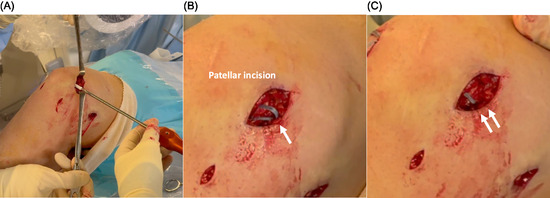
First stage of staged tension adjustment of the medial patellofemoral ligament: fixation of the proximal bundle (right knee, medial views). A 5 × 20‐mm BIOSURE screw (Smith & Nephew Endoscopy) is inserted into the proximal tunnel to secure the ULTRATAPE (A). At this stage, the double‐bundle construct remains untensioned, allowing staged adjustment. By pulling the free end of the distal ULTRATAPE, further adjustment of graft tension can be achieved (B,C). (Single white arrow, untensioned ULTRATAPE; double white arrows, tensioned ULTRATAPE.)

**FIGURE 7:**
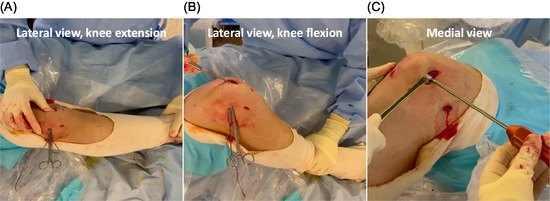
Second stage of staged tension adjustment of the medial patellofemoral ligament: tension adjustment and fixation of the distal bundle (right knee, lateral and medial views). The ULTRATAPE emerging laterally from the distal tunnel is temporarily clamped with a Kocher clamp. The knee is then subjected to extension and flexion to assess patellar stability, ensuring that no over‐tightening occurs, especially beyond 60° of flexion (A,B). If tension is suboptimal, the clamp position is adjusted. Finally, a second 5 × 20‐mm BIOSURE screw is inserted into the distal tunnel to secure the ULTRATAPE at the desired tension (C).

A 5 × 20‐mm BIOSURE REGENESORB Interference Screw (Smith & Nephew Endoscopy) is inserted into the proximal tunnel to secure the ULTRATAPE (Figure 6A). At this stage, the double‐bundle construct remains untensioned, allowing for a staged adjustment. By pulling the free end of the distal ULTRATAPE, further adjustment of the graft tension can be achieved (Figure 6B,C). The strand emerging laterally from the distal tunnel is temporarily clamped using a Kocher clamp. The knee is then taken through a full range of extension and flexion to assess patellar stability, confirming the absence of dislocation in full extension and avoiding excessive graft tension, particularly beyond 60° of flexion (Figure 7A,B). If the tension is suboptimal, the clamp position is adjusted. Finally, a second 5 × 20‐mm BIOSURE REGENESORB Interference Screw is inserted into the distal tunnel to secure the ULTRATAPE at the desired tension (Figure 7C).

### Confirmation of MPFL Tension Under Arthroscopy

**FIGURE 8:**
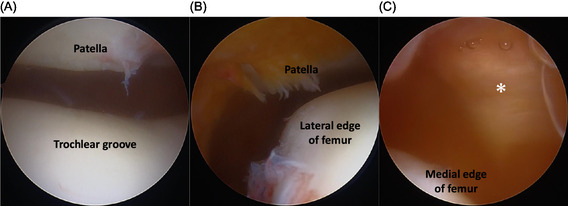
Postoperative arthroscopic evaluation (right knee, anterolateral portal views). Improved patellofemoral congruence (A), correction of lateral patellar displacement (B), and appropriate tension in the reconstructed medial patellofemoral ligament (white asterisk) (C) can be confirmed.

Arthroscopy is performed to confirm graft tension. Adequate patellar tracking and stability are verified and compared with the preoperative state to ensure appropriate tension in the reconstructed MPFL (Figure 8A‐C).

### Postoperative Rehabilitation

Patients are allowed full weight bearing with crutches on the first postoperative day. Early ROM and quadriceps control exercises, including patellar setting and straight leg raising, are initiated immediately. Jogging is permitted at 2 months, with return to sports activities once neuromuscular control and function are restored.

## DISCUSSION

We describe a double‐bundle artificial MPFL reconstruction technique using a femoral suture‐sliding anchor and patellar staged tension adjustment. The key advantage of this method is its ability to fine‐tune the graft tension intraoperatively, which is one of the most critical issues in artificial ligament reconstruction. Compared with autografts, artificial grafts provide strong initial fixation, but they lack elasticity, and their stiffness carries a risk of over‐tensioning, which may lead to anterior knee pain, delayed rehabilitation, or degenerative changes. Our staged fixation technique allows intraoperative adjustment of the double‐bundle MPFL, thereby reducing the risk of over‐constraint. The advantages and disadvantages of this technique are summarized in Table 1.

**TABLE 1:** Advantages and Disadvantages

Advantages	Disadvantages
Allows staged intraoperative tension adjustment, reducing risk of over‐constraint	Requires two patellar tunnels, which may increase risk of patellar fracture
Reproduces fan‐shaped native medial patellofemoral ligament anatomy with double‐bundle construct	Use of artificial ligament raises concerns about long‐term biological response
Femoral suture‐sliding anchor permits postoperative mobility, potentially relieving excessive tension	Not suitable for skeletally immature patients or those with severe trochlear dysplasia
Provides reproducible and minimally invasive fixation	
Can be modified for small patella (proximal [one‐quarter] tunnel omitted, fixed to the periosteum)	

Previous studies have emphasized the importance of the graft fixation angle and tension in MPFL reconstruction. Previous reports stressed that over‐tensioning of the suture tape leads to knee pain and need for revision surgery.^7^, ^8^ Nomura and Inoue^9^ recommended fixation at greater flexion angles (60°‐90°) when using synthetic grafts to decrease the medial patellofemoral contact pressure. Our staged tension technique does not rely solely on a specific flexion angle at the time of fixation, because the suture‐sliding anchor and sequential patellar fixation allow intraoperative adjustment across the full ROM. This adjustability may overcome one of the main limitations of the previously reported artificial ligament reconstructions. The key surgical pearls and potential pitfalls of this technique are summarized in Table 2.

**TABLE 2:** Pearls and Pitfalls

Pearls	Pitfalls
Indicated for patients with recurrent patellar instability without severe bony abnormalities	Malposition of the femoral fixation point can lead to graft failure
Use of femoral suture‐sliding anchor allows staged tension adjustment during surgery	Excessive tunnel size or misplacement may predispose to patellar fracture
Assess patellar tracking through full range of motion before final fixation	Reliance on synthetic grafts makes long‐term durability uncertain
When patella is small, omit proximal 1/4 tunnel and fix ULTRATAPE to periosteum	
Confirm final graft tension arthroscopically to ensure stability without over‐constraint	

Another notable advantage of this technique is its applicability, even in cases of postoperative over‐tensioning. Because the femoral suture‐sliding anchor allows smooth gliding of the ULTRATAPE, graft mobility may compensate for residual over‐constraint, which is not possible with rigid fixation methods, such as interference screws or buttons.

This procedure has some limitations. First, as with all techniques using synthetic materials, the long‐term biological response and durability remain uncertain, and further clinical follow‐up is required to clarify its safety. Second, the creation of two patellar tunnels carries a theoretical risk of patellar fracture, albeit this risk was minimized by carefully positioning the tunnels under fluoroscopic guidance. In cases where the patella is small, the proximal (one‐quarter) bone tunnel may be omitted, and ULTRATAPE can be fixed by suturing it to the proximal periosteum of the patella. In this modification, the initial fixation of the proximal bundle in the double‐bundle technique is substituted by periosteal suturing, while maintaining stable distal fixation with a BIOSURE screw. This modification reduces the risk of iatrogenic patellar fracture while maintaining stable fixation.

In summary, double‐bundle artificial MPFL reconstruction using a femoral suture‐sliding anchor and staged patellar fixation offers a reproducible method to adjust graft tension, reduce the risk of over‐constraint, and better restore the anatomical configuration of the native MPFL. Further clinical studies with longer follow‐up periods are required to confirm the long‐term safety and outcomes of this technique.

## DISCLOSURES

The authors (R.S., T.O., T.N., T.H., K.K., M.N., H.M.) declare that they have no known competing financial interests or personal relationships that could have appeared to influence the work reported in this paper.
